# 3-(2-Acetamido­phen­yl)sydnone

**DOI:** 10.1107/S1600536809005066

**Published:** 2009-02-21

**Authors:** David A. Grossie, Kenneth Turnbull, Sandra Felix-Balderrama, Shravanthi Raghavapuram

**Affiliations:** a Department of Chemistry, Wright State University, 3640 Colonel Glenn Hwy, Dayton, Ohio 45435, USA

## Abstract

Sydnones are unusual mesoionic compounds containing a five-membered heterocyclic ring. Generally for stability, substitution at the N-3 position by an aromatic fragment is necessary. In the title compound, C_10_H_9_N_3_O_3_, the aromatic substitutent is 2-acetamido­phenyl. The two planar ring fragments are twisted relative to one another, with a inter­planar angle of 63.13 (5)°. The mol­ecules are packed into the unit cell *via* π–π inter­actions between the phenyl rings [inter­planar separation = 3.4182 (4) Å] and between the sydnone rings [inter­planar separation = 3.2095 (4) Å]. N—H⋯O and C—H⋯O hydrogen bonding is also found inter­nally and externally to the mol­ecule.

## Related literature

For more information on the sydnone family of compounds, see: Ohta & Kato (1969 ▶). For the synthesis and structural information, see: Grossie *et al.* (1992 ▶, 2001 ▶, 2007 ▶); Riddle *et al.* 2004*a* ▶,*b* ▶,*c* ▶; Hope & Thiessen (1968 ▶); Hodson & Turnbull (1985 ▶); Baker & Ollis (1957 ▶). For a description of the Cambridge Structural Database, see: Allen (2002 ▶). For related literature, see: Kier & Roche (1966 ▶); Matsunaga (1957 ▶); Ollis & Ramsden (1976 ▶).
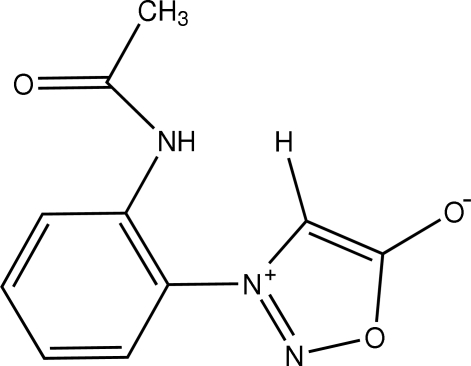


## Experimental

### 

#### Crystal data

C_10_H_9_N_3_O_3_
*M* *_r_* = 219.20
Monoclinic, 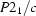

*a* = 7.7348 (4) Å
*b* = 13.7212 (7) Å
*c* = 9.6698 (5) Å
β = 106.083 (1)°
*V* = 986.10 (9) Å^3^
*Z* = 4
Mo *K*α radiation
μ = 0.11 mm^−1^
*T* = 173 K
0.45 × 0.40 × 0.26 mm

#### Data collection

Bruker SMART APEXII diffractometer
Absorption correction: multi-scan (*SADABS*; Bruker, 2003 ▶) *T*_min_ = 0.895, *T*_max_ = 0.970
8741 measured reflections
3053 independent reflections
2711 reflections with *I* > 2σ(*I*)
*R*_int_ = 0.017

#### Refinement

*R*[*F* ^2^ > 2σ(*F* ^2^)] = 0.038
*wR*(*F* ^2^) = 0.108
*S* = 1.05
3053 reflections
150 parameters
H atoms treated by a mixture of independent and constrained refinement
Δρ_max_ = 0.44 e Å^−3^
Δρ_min_ = −0.24 e Å^−3^

### 

Data collection: *SMART* (Bruker, 2003 ▶); cell refinement: *SAINT-Plus* (Bruker, 2003 ▶); data reduction: *SAINT-Plus*; program(s) used to solve structure: *SHELXS97* (Sheldrick, 2008 ▶); program(s) used to refine structure: *SHELXL97* (Sheldrick, 2008 ▶); molecular graphics: *Mercury* (Macrae *et al.*, 2008 ▶) and *OSCAIL X*, (McArdle, 2008 ▶); software used to prepare material for publication: *enCIFer* (Allen *et al.*, 2004 ▶), *publCIF* (Westrip, 2009 ▶) and *PLATON* (Spek, 2009 ▶).

## Supplementary Material

Crystal structure: contains datablocks I, global. DOI: 10.1107/S1600536809005066/rk2130sup1.cif

Structure factors: contains datablocks I. DOI: 10.1107/S1600536809005066/rk2130Isup2.hkl

Additional supplementary materials:  crystallographic information; 3D view; checkCIF report

## Figures and Tables

**Table 1:** Hydrogen-bond geometry (Å, °)

*D*—H⋯*A*	*D*—H	H⋯*A*	*D*⋯*A*	*D*—H⋯*A*
N12—H12⋯O17*A*^i^	0.876 (15)	2.056 (15)	2.9272 (10)	173.2 (14)
C4—H4⋯O5^ii^	0.96	2.28	3.1860 (12)	156
C13—H13⋯O17*A*	0.96	2.29	2.8587 (14)	117
C15—H15⋯O5^iii^	0.96	2.41	3.3612 (14)	173
C16—H16⋯O5^iv^	0.96	2.57	3.4700 (13)	157
C18—H18*B*⋯O17*A*^i^	0.98	2.54	3.3201 (12)	136

Symmetry codes: (i) 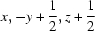; (ii) 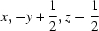; (iii) 
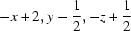
; (iv) 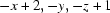.

**Fig. 1.**
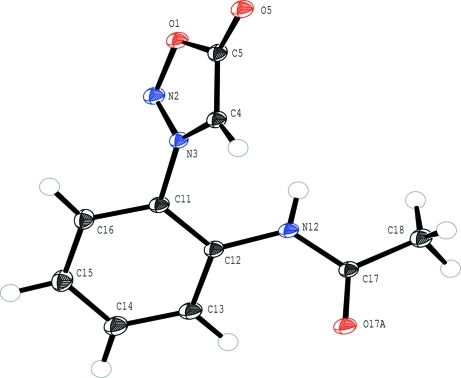
Molecular structure of title compound with the atom numbering scheme. The displacement ellipsoids are drawn at 50% probability level. The H atoms are presented as a small spheres of arbitrary radius.

## supplementary crystallographic information

### Comment

Sydnones are the most widely studied members of the general class of mesoionic compounds (Ohta & Kato, 1969). They are the products of dehydration of *N*–nitroso–α–aminoacids (Hope & Thiessen, 1968), and they undergo facile electrophilic aromatic subtitution on the sydnone ring. The selectivity on the sydnone ring (and not the aryl ring) can be attributed to the activated nature of the sydnone ring (Matsunaga, 1957) and its deactivating effect upon the attached aryl ring (the N3 position bears a considerable fractional positive charge (Kier & Roche, 1966)).

The 3–(2–acetamidophenyl)sydnone was synthesized to investigate its bromination and to probe the parameters controlling the site of electrophilic attack, both from mechanistic and synthetic standpoints. The sydnone ring is found to be planar. The ring bond distances O–N, N–N, N–C and C–C are similar to those of related compounds. However, the C—O bond distance of 1.4158 (12)Å, is longer than that of the C—O bond in a furane ring. As mentioned in previous paper (Hodson & Turnbull, 1985), the exocyclic C═O distance (1.2181 (12)Å) does not support the formulation of Baker & Ollis (1957), which involves the delocalization of a positive charge on the ring, and a negative charge on the exocyclic oxygen.

The molecules pack along the body diagonal within the unit cell, in symmetry related pairs with the phenyl rings lying parallel to each other, separated by a distance of 3.4182 (4)Å. The pairs of molecules are further paired through interaction of the sydnone rings in adjacent molecules, which are positioned parallel to each other at a distance of 3.2095 (4)Å. The molecules are connected laterally through hydrogen bonding between the sydnone and acetamide O atoms and phenyl, and amide H atoms. Hydrogen bond parameters are tabulated in Table 1.

As expected the sydnone ring was similar in metrical parameters to sydnone structures previously determined, in this laboratory (Grossie *et al.*, 1992, 2001, 2007; Riddle *et al.* 2004*a*,*b*,*c*) and as found in the Cambridge Structural Database vers. 5.30 (Allen, 2002).

### Experimental

3–(2–Aminophenyl)sydnone (0.5 g, 2.82 mmol) was dissolved in acetic anhydride (10 ml) and stirred for 20 h. Evaporation on standing overnight gave a light yellow oil which was crystallized from methylene chloride/petroleum ether to give 3–(2–acetamidophenyl)sydnone (0.42 g, 68%), mp 349–351 K; IR–spectra: 3310, 3290 (N—H str), 3130 (sydnone C—H str), 1740 (sydnone C═O str) cm^-1^. The ^1^H NMR (CDCl_3_): δ 2.0 (s, 3H), 6.83 (s, 1H), 7.65 (m, 4H), 9.65 (s, 1H). Anal. Calcd. for C_10_H_9_N_3_O_3_: C, 54.79; H, 4.11; N, 19.18. Found; C, 54.90; H, 4.17; N, 18.89.

### Refinement

The amide H atom was located in a different Fourier map and refined with an isotropic displacement parameter. The positional parameters were allow to refine freely. The methyl and benzene H atoms were included in geometrically calculated positions, with C—H distances of 0.98Å and 0.96Å, respectively, and *U*_iso_(H) = 1.3*U*_eq_(C).

### Crystal data

**Table:**

C_10_H_9_N_3_O_3_	*F*(000) = 456
*M**_r_* = 219.20	*D*_x_ = 1.477 Mg m^−^^3^
Monoclinic, *P*2_1_/*c*	Mo *K*α radiation, λ = 0.71073 Å
Hall symbol: -P 2ybc	Cell parameters from 4272 reflections
*a* = 7.7348 (4) Å	θ = 2.7–31.7°
*b* = 13.7212 (7) Å	µ = 0.11 mm^−^^1^
*c* = 9.6698 (5) Å	*T* = 173 K
β = 106.083 (1)°	Block, white
*V* = 986.10 (9) Å^3^	0.45 × 0.40 × 0.26 mm
*Z* = 4	

### Data collection

**Table:**

Bruker SMART APEXII diffractometer	3053 independent reflections
Radiation source: Fine–focus sealed tube	2711 reflections with *I* > 2σ(*I*)
Graphite	*R*_int_ = 0.017
ω scans	θ_max_ = 32.0°, θ_min_ = 2.7°
Absorption correction: multi-scan (*SADABS*; Bruker, 2003)	*h* = −11→11
*T*_min_ = 0.895, *T*_max_ = 0.970	*k* = −19→14
8741 measured reflections	*l* = −14→14

### Refinement

**Table:**

Refinement on *F*^2^	Primary atom site location: Direct
Least-squares matrix: Full	Secondary atom site location: Difmap
*R*[*F*^2^ > 2σ(*F*^2^)] = 0.038	Hydrogen site location: Geom
*wR*(*F*^2^) = 0.108	H atoms treated by a mixture of independent and constrained refinement
*S* = 1.05	*w* = 1/[σ^2^(*F*_o_^2^) + (0.058*P*)^2^ + 0.315*P*] where *P* = (*F*_o_^2^ + 2*F*_c_^2^)/3
3053 reflections	(Δ/σ)_max_ < 0.001
150 parameters	Δρ_max_ = 0.44 e Å^−^^3^
0 restraints	Δρ_min_ = −0.24 e Å^−^^3^

### Special details

**Table:**

Geometry. All s.u.'s (except the s.u. in the dihedral angle between two l.s. planes) are estimated using the full covariance matrix. The cell s.u.'s are taken into account individually in the estimation of s.u.'s in distances, angles and torsion angles; correlations between s.u.'s in cell parameters are only used when they are defined by crystal symmetry. An approximate (isotropic) treatment of cell s.u.'s is used for estimating s.u.'s involving l.s. planes. Least–Squares Planes. Sydnone Ring. Defining atoms: O1 0.004 (1)Å, N2 -0.003 (1)Å, N3 0.000 (1)Å, C4 0.003 (1)Å, C5 -0.004 (1)Å; other atoms: O5 0.002 (1)Å, C11 0.081 (1)Å. Phenyl Ring. Defining atoms: C11 0.009 (1)Å, C12 -0.004 (1)Å, C13 -0.003 (1)Å, C14 0.006 (1)Å, C15 -0.002 (1)Å, C16 -0.006 (1)Å; other atoms: O17A 0.710 (1)Å, N12 -0.120 (1)Å, C17 0.159 (1)Å, C18 -0.233 (1)Å.
Refinement. Refinement of *F*^2^ against ALL reflections. The weighted *R*–factor *wR* and goodness of fit *S* are based on\ *F*^2^, conventional *R*–factors *R* are based on *F*, with *F* set to zero for negative *F*^2^. The threshold expression of *F*^2^ > σ(*F*^2^) is used only for calculating *R*–factors(gt) *etc*. and is not relevant to the choice of reflections for refinement. *R*–factors based on *F*^2^ are statistically about twice as large as those based on *F*, and *R*–factors based on ALL data will be even larger.

### Fractional atomic coordinates and isotropic or equivalent isotropic displacement parameters (Å^2^)

**Table:**

	*x*	*y*	*z*	*U*_iso_*/*U*_eq_	
O1	0.84105 (9)	0.07745 (5)	0.51445 (7)	0.01803 (16)	
N3	0.82573 (10)	0.08267 (6)	0.29241 (8)	0.01461 (16)	
N2	0.76980 (11)	0.03010 (6)	0.38528 (9)	0.01838 (18)	
C4	0.92672 (12)	0.16064 (7)	0.34726 (10)	0.01603 (18)	
H4	0.9791	0.2067	0.2959	0.021*	
C5	0.94005 (12)	0.16089 (7)	0.49604 (10)	0.01573 (18)	
O5	1.01261 (10)	0.21147 (6)	0.59934 (8)	0.01993 (16)	
C11	0.78190 (12)	0.04762 (7)	0.14601 (9)	0.01569 (18)	
C12	0.67371 (12)	0.10348 (7)	0.03402 (10)	0.01569 (18)	
N12	0.59485 (11)	0.19068 (6)	0.06408 (8)	0.01579 (16)	
H12	0.579 (2)	0.1987 (11)	0.1492 (16)	0.024 (3)*	
C13	0.63770 (14)	0.06464 (8)	−0.10516 (10)	0.0210 (2)	
H13	0.5644	0.1005	−0.1853	0.027*	
C14	0.70713 (15)	−0.02531 (8)	−0.12794 (11)	0.0231 (2)	
H14	0.6820	−0.0503	−0.2242	0.030*	
C15	0.81211 (14)	−0.08014 (8)	−0.01474 (11)	0.0219 (2)	
H15	0.8582	−0.1425	−0.0322	0.028*	
C16	0.84924 (13)	−0.04322 (7)	0.12408 (11)	0.01917 (19)	
H16	0.9207	−0.0800	0.2040	0.025*	
C17	0.52287 (12)	0.26254 (7)	−0.03227 (10)	0.01474 (17)	
O17A	0.53832 (10)	0.26491 (6)	−0.15559 (7)	0.02026 (16)	
C18	0.42424 (13)	0.34120 (8)	0.02328 (10)	0.01933 (19)	
H18A	0.5042	0.3972	0.0544	0.025*	
H18B	0.3858	0.3162	0.1050	0.025*	
H18C	0.3184	0.3615	−0.0534	0.025*	

### Atomic displacement parameters (Å^2^)

**Table:**

	*U*^11^	*U*^22^	*U*^33^	*U*^12^	*U*^13^	*U*^23^
O1	0.0216 (3)	0.0193 (4)	0.0135 (3)	−0.0025 (3)	0.0055 (2)	0.0016 (2)
N3	0.0158 (3)	0.0144 (4)	0.0133 (3)	0.0005 (3)	0.0036 (3)	0.0016 (3)
N2	0.0218 (4)	0.0187 (4)	0.0148 (3)	−0.0033 (3)	0.0053 (3)	0.0015 (3)
C4	0.0183 (4)	0.0157 (4)	0.0143 (4)	−0.0019 (3)	0.0049 (3)	0.0007 (3)
C5	0.0166 (4)	0.0155 (4)	0.0155 (4)	0.0016 (3)	0.0051 (3)	0.0020 (3)
O5	0.0242 (3)	0.0201 (4)	0.0155 (3)	−0.0006 (3)	0.0055 (3)	−0.0021 (3)
C11	0.0169 (4)	0.0170 (4)	0.0130 (4)	−0.0004 (3)	0.0038 (3)	−0.0013 (3)
C12	0.0174 (4)	0.0159 (4)	0.0137 (4)	0.0007 (3)	0.0043 (3)	−0.0008 (3)
N12	0.0201 (4)	0.0167 (4)	0.0108 (3)	0.0029 (3)	0.0047 (3)	0.0003 (3)
C13	0.0250 (5)	0.0219 (5)	0.0144 (4)	0.0030 (4)	0.0027 (3)	−0.0028 (3)
C14	0.0282 (5)	0.0223 (5)	0.0186 (4)	0.0013 (4)	0.0060 (4)	−0.0062 (4)
C15	0.0243 (5)	0.0175 (5)	0.0244 (5)	0.0015 (3)	0.0074 (4)	−0.0042 (4)
C16	0.0201 (4)	0.0160 (5)	0.0208 (4)	0.0016 (3)	0.0047 (3)	0.0004 (3)
C17	0.0148 (4)	0.0163 (4)	0.0130 (4)	−0.0016 (3)	0.0037 (3)	0.0000 (3)
O17A	0.0294 (4)	0.0199 (4)	0.0130 (3)	−0.0011 (3)	0.0084 (3)	0.0009 (3)
C18	0.0219 (4)	0.0201 (5)	0.0174 (4)	0.0059 (3)	0.0078 (3)	0.0027 (3)

### Geometric parameters (Å, °)

**Table:**

O1—N2	1.3801 (10)	N12—H12	0.871 (15)
O1—C5	1.4158 (12)	C13—C14	1.3878 (15)
N3—N2	1.3150 (11)	C13—H13	0.9600
N3—C4	1.3440 (12)	C14—C15	1.3897 (15)
N3—C11	1.4437 (12)	C14—H14	0.9600
C4—C5	1.4136 (12)	C15—C16	1.3883 (14)
C4—H4	0.9600	C15—H15	0.9600
C5—O5	1.2181 (12)	C16—H16	0.9600
C11—C16	1.3897 (14)	C17—O17A	1.2314 (11)
C11—C12	1.3994 (13)	C17—C18	1.5041 (14)
C12—C13	1.4018 (13)	C18—H18A	0.9800
C12—N12	1.4093 (12)	C18—H18B	0.9800
N12—C17	1.3649 (12)	C18—H18C	0.9800
			
N2—O1—C5	111.17 (7)	C14—C13—H13	119.7
N2—N3—C4	115.61 (8)	C12—C13—H13	119.7
N2—N3—C11	117.04 (8)	C13—C14—C15	121.59 (9)
C4—N3—C11	127.23 (8)	C13—C14—H14	119.2
N3—N2—O1	103.62 (7)	C15—C14—H14	119.2
N3—C4—C5	105.91 (8)	C16—C15—C14	118.90 (9)
N3—C4—H4	127.0	C16—C15—H15	120.6
C5—C4—H4	127.0	C14—C15—H15	120.6
O5—C5—C4	136.32 (9)	C15—C16—C11	119.27 (9)
O5—C5—O1	120.00 (8)	C15—C16—H16	120.4
C4—C5—O1	103.68 (8)	C11—C16—H16	120.4
C16—C11—C12	122.84 (9)	O17A—C17—N12	123.33 (9)
C16—C11—N3	116.89 (8)	O17A—C17—C18	121.46 (9)
C12—C11—N3	120.26 (8)	N12—C17—C18	115.21 (8)
C11—C12—C13	116.86 (9)	C17—C18—H18A	109.5
C11—C12—N12	120.37 (8)	C17—C18—H18B	109.5
C13—C12—N12	122.58 (8)	H18A—C18—H18B	109.5
C17—N12—C12	126.20 (8)	C17—C18—H18C	109.5
C17—N12—H12	114.7 (10)	H18A—C18—H18C	109.5
C12—N12—H12	118.8 (10)	H18B—C18—H18C	109.5
C14—C13—C12	120.52 (9)		
			
C4—N3—N2—O1	−0.25 (11)	N3—C11—C12—C13	179.79 (9)
C11—N3—N2—O1	176.13 (7)	C16—C11—C12—N12	173.82 (9)
C5—O1—N2—N3	0.65 (10)	N3—C11—C12—N12	−5.04 (14)
N2—N3—C4—C5	−0.24 (11)	C11—C12—N12—C17	163.84 (9)
C11—N3—C4—C5	−176.19 (8)	C13—C12—N12—C17	−21.28 (15)
N3—C4—C5—O5	179.52 (11)	C11—C12—C13—C14	0.22 (15)
N3—C4—C5—O1	0.61 (10)	N12—C12—C13—C14	−174.83 (10)
N2—O1—C5—O5	−179.93 (8)	C12—C13—C14—C15	0.73 (17)
N2—O1—C5—C4	−0.80 (10)	C13—C14—C15—C16	−0.59 (17)
N2—N3—C11—C16	−60.99 (12)	C14—C15—C16—C11	−0.50 (16)
C4—N3—C11—C16	114.91 (11)	C12—C11—C16—C15	1.51 (15)
N2—N3—C11—C12	117.94 (10)	N3—C11—C16—C15	−179.60 (9)
C4—N3—C11—C12	−66.16 (13)	C12—N12—C17—O17A	−9.29 (15)
C16—C11—C12—C13	−1.34 (15)	C12—N12—C17—C18	171.74 (9)

### Hydrogen-bond geometry (Å, °)

**Table:**

*D*—H···*A*	*D*—H	H···*A*	*D*···*A*	*D*—H···*A*
N12—H12···O17A^i^	0.876 (15)	2.056 (15)	2.9272 (10)	173.2 (14)
C4—H4···O5^ii^	0.96	2.28	3.1860 (12)	156
C13—H13···O17A	0.96	2.29	2.8587 (14)	117
C15—H15···O5^iii^	0.96	2.41	3.3612 (14)	173
C16—H16···O5^iv^	0.96	2.57	3.4700 (13)	157
C18—H18B···O17A^i^	0.98	2.54	3.3201 (12)	136

Symmetry codes: (i) *x*, −*y*+1/2, *z*+1/2; (ii) *x*, −*y*+1/2, *z*−1/2;  (iii) −*x*+2, *y*−1/2, −*z*+1/2; (iv) −*x*+2, −*y*, −*z*+1.
